# Fermentation of Wheat Bran and Whey Permeate by Mono-Cultures of *Lacticaseibacillus rhamnosus* Strains and Co-culture With Yeast Enhances Bioactive Properties

**DOI:** 10.3389/fbioe.2020.00956

**Published:** 2020-08-07

**Authors:** Annalisse Bertsch, Denis Roy, Gisèle LaPointe

**Affiliations:** ^1^Department of Food Science, Laval University, Quebec City, QC, Canada; ^2^Institute of Nutrition and Functional Foods (INAF), Laval University, Quebec City, QC, Canada; ^3^Department of Food Science, University of Guelph, Guelph, ON, Canada

**Keywords:** coculture, lactic acid bacteria, yeast, wheat bran, bioaccessibility, phenolic compounds

## Abstract

The aim of this work was to obtain a bioingredient (BI) with bioactive properties through the solid fermentation of a wheat bran-whey permeate (WB/WP) mixture with three strains of *Lacticaseibacillus rhamnosus* (R0011, ATCC 9595, and RW-9595M) in mono or co-culture with *Saccharomyces cerevisiae*. The choice of these strains was based on their capacity to produce the same exopolysaccharide (EPS), but at different yields. The solid fermentation of WB/WP revealed a similar growth pattern, sugar utilization and metabolite production between strains and types of culture. Lactic acid, soluble protein, free amino acid and phenolic compound content in BI were compared to NFWB. Water soluble polysaccharides (including EPS) were significantly increased in co-culture for (44%) ATCC 9595, (40%) R0011 and (27%) RW-9595M. The amount of bound Total Phenolic Content (TPC) as well as the antioxidant activity in BI were higher after fermentation. The free phenolic acid content was higher after fermentation with ATCC 9595 (53–59%), RW-9595M (45–46%), and R0011 (29–39%) compared to non-fermented NFWB. Fermentation by these strains increased the amounts of free caffeic acid and 4-hydroxybenzoic acid in both types of culture. The bound phenolic acid content was enhanced in co-culture for the BI obtained from the highest EPS producer strain RW-9595M which was 30% higher than NFWB. After *in vitro* digestion, bioaccessibility of free total phenolic acids was improved by more than 40% in BI compared to NFWB. The co-culture increased recovery of TPC (%) and antioxidant activity compared to monoculture for the strains in digested product. In contrast, the recovery of bound total phenolic acids in co-culture was 33 and 38% lower when compared to monoculture for R0011 and RW-9595M. Our findings provide new insights into the impact of LAB/yeast co-culture on the bioactive properties of fermented wheat bran.

## Introduction

Cereals have increasingly been used in fermentation to produce fiber-enhanced beverages, as well as potential prebiotic sources in probiotic dairy products and bakery products (Stevenson et al., 2012; Onipe et al., 2015; Russo et al., 2016). Wheat bran (WB) has attracted much interest because it represents the most important milling by-product of grain cereals with a production of approximately 150 million tons per year. Wheat bran is a complex substrate composed mainly of dietary fiber, proteins and starch (Anson et al., 2011; Onipe et al., 2015). It contains vitamins, minerals and bioactive molecules, such as low-molecular weight phenolic acid compounds including p-coumaric and mainly ferulic acid (FA), which have shown antioxidant activity (Anson et al., 2011, Anson et al., 2012; Laddomada et al., 2015). In wheat bran, the phenolic compounds can be found in free form or linked to the structural components of plants (cellulose, arabinoxylan, and proteins) through ester bonds or covalently bound (Anson et al., 2012). Fermentation can improve the nutritional, functional, and sensorial properties of wheat bran (Poutanen et al., 2009; Katina et al., 2012). Yeast fermentation of wheat bran increased the level of folates (1.62-fold), free ferulic acid (5.5-fold), and soluble arabinoxylan (1.54-fold) (Katina et al., 2012). Bio-processing causes hydrolysis and solubilization of proteins and fibers allowing the delivery of bioactive and potentially protective compounds when consumed (Coda et al., 2014; Verni et al., 2019).

During fermentation, changes in temperature and pH of the medium can influence the action of both endogenous and bacterial enzymes. Grain constituents are modified by hydrolysis of water insoluble arabinoxylan causing cleavage in the backbone structure generating arabinoxylan-oligosaccharide (AXOS), which enhances total phenolic content and antioxidant activities principally by releasing free ferulic acid (Anson et al., 2012; Coda et al., 2014). However, the bioavailability of the wheat bran fractions depends on the proportion of the compounds in the food matrix, as well as their release and possible absorption by the intestine (bioaccessibility). The free phenolic compounds are mainly hydrolyzed and a small portion is absorbed in the upper intestinal tract of humans. Conversely, the insoluble, bound compounds represent the main fraction (83%) of phenolic compounds that pass through the small intestine without absorption (Cömert and Gökmen, 2017). Thus, they can undergo fermentation by the colon microbiota, which may result in metabolites with greater biological activity (Valdés et al., 2015).

Lactic acid bacteria (LAB) have been co-cultured with yeast for fermentation of bran (Coda et al., 2014; Zhao et al., 2017). Strains of *Lacticaseibacillus rhamnosus* or *Saccharomyces cerevisiae* in monoculture were able to enhance levels of antioxidant activity in cereals (Dordevic et al., 2010). The total phenolic content (TPC) was increased in wheat bran fermented by these two microorganism in co-culture more than in monoculture (Zhao et al., 2017). To promote microbial growth, several authors reported the supplementation of cereal-based medium with sucrose, raffinose or glucose (Kajala et al., 2016; Xu et al., 2017). Similarly, whey permeate (WP) has been used as a carbon source (lactose) in LAB fermentations (Macedo et al., 2002; Bergmaier et al., 2003). Therefore, the combination of both cereal and WP represents a promising source for the creation of innovative fermented bioproducts (Slukovà et al., 2016). Furthermore, *Lacticaseibacillus* spp. are able to produce exopolysaccharides (EPS) impacting texture and viscosity, while also potentially exhibiting prebiotic or antioxidant properties that can improve the bioactivity of fermented cereal products (Cheirsilp et al., 2003; Polak-Berecka et al., 2015; Salazar et al., 2016). Several studies reported that lactate consumption by the yeast can probably enhance the production of kefiran or bacteriocins (nisin) under controlled pH in LAB–yeast co-culture (Minekus et al., 2014; Chanos and Mygind, 2016). Despite the fact that *S. cerevisiae* and LAB co-exist in cereal fermented products such as sourdough, improved understanding of their interactions and their effect on the bioaccessibility of phenolic acid compounds is still needed.

Static *in vitro* gastrointestinal digestion systems can replace animal and human models for rapidly screening food and bioingredients; additionally *in vitro* practices are faster and less expensive than *in vivo* approaches, while avoiding some ethical issues (Minekus et al., 2014). In food technology, the bioaccessibility and bioavailability of phenolic acid compounds can be estimated using *in vitro* simulated gastrointestinal digestion (Motilva et al., 2015; Zeng et al., 2016). However, little information has been published on these properties of bioactive compounds in ingredients obtained from fermented wheat bran subjected to *in vitro* gastrointestinal digestion. Therefore, this study focused on the effect of mono or co-culture as well as the presence of EPS on the bioaccessibility of phenolic acid compounds of fermented wheat bran. The first aim was to obtain bioingredients with bioactive properties through the fermentation of a wheat bran-whey permeate (WB/WP) mixture by 3 strains of *L. rhamnosus* in mono or co-culture with *S. cerevisiae.* The second aim was to investigate the influence of the type of culture on chemical composition of the bioingredients. The third aim was to evaluate the content of free and bound total phenolic compounds, antioxidant activity and the phenolic acid profile in the bioingredients. Finally, the bio-products were submitted to *in vitro* digestion in order to relate the properties to bioaccessibility and recovery of phenolic compounds.

## Materials and Methods

### Bacteria and Culture Conditions

*Lacticaseibacillus rhamnosus* ATCC 9595 was obtained from the American Type Culture Collection (ATCC; Manassas, VA). *L. rhamnosus* RW-9595M (variant of ATCC 9595) and *L. rhamnosus* R0011 (also known as R or Rosell-11, from Lallemand Health Solutions) were obtained from our lactic acid bacteria culture collection (Université Laval, Quebec, Canada). These strains produced different quantities of the same EPS and their production was increased in co-culture with yeast in a previous study (van Calsteren et al., 2002; Bertsch et al., 2019). The stock culture was maintained at −80°C in 6% (v/v) rehydrated skim milk and 10% (v/v) glycerol. The commercial (Lallemand Instaferm Gold Instant dry yeast) baker’s yeast (Lallemand Instaferm) *S. cerevisiae* was grown in YEPAL medium (1% yeast extract, 2% peptone, and 1% lactic acid) at pH 5.0. This medium was used to adapt the yeast to lactic acid because the yeast can utilize lactic acid but cannot utilize lactose, which is the major carbon source in the WB/WP medium (Yamasaki-Yashiki et al., 2017).

Commercial Wheat Bran had a mean particle size of 750 μm and whey permeate WP was obtained from Agropur, Canada. Whey permeate powder was added to water to give a final concentration of 5% (w/w) and was autoclaved (121°C, 15 min) (Bergmaier et al., 2003). Analytical grade chemicals, solvents, and reagents were used unless specified otherwise.

### Fermentation

The stock cultures of *L. rhamnosus* were incubated at 37°C for 8 h in 10 mL of MRS-L (MRS supplemented with 20 g/l lactose). Next, 2% (v/v) was added to 50 mL of WP supplemented with yeast extract, followed by incubation at 37°C for 12 h. A yeast colony from YEPAL agar was added to 20 mL of YEPAL broth, which was incubated at 30°C in a shaker at 180 rpm for 24 h.

A mixture of WB/WP (14:86% w/v) was inoculated with 3.5% cell suspension of *L. rhamnosus* for the monoculture or 1.75% of *L. rhamnosus* + 1.75% of yeast for the co-culture (1:1 ratio). Solid wheat bran fermentations were carried out in mono (lactic acid bacteria) or co-culture (lactic acid bacteria and baker’s yeast) at 35°C without agitation (Table 1). A control (WB) sample containing a mixture of cereal and WP was prepared. All fermentations were sampled after 0, 3, 6, 9, 12, 15, and 24 h of incubation to monitor bacterial growth (by plate counts), pH, lactose consumption, and metabolite production (lactic acid, acetic acid). All fermentations were performed in triplicate.

**TABLE 1:** Bioprocessing combinations of the wheat bran/whey permeate (WB/WP) mixture and strains* to obtain the fermented bioingredients and *in vitro* product of digestion.

Strain	Type of culture	Bioingredient
*Lacticaseibacillus rhamnosus* R0011	M	LR11M
*Lacticaseibacillus rhamnosus* R0011 + *S. cerevisiae*	C	LR11C
*Lacticaseibacillus rhamnosus* ATCC 9595	M	LR95M
*Lacticaseibacillus rhamnosus* ATCC 9595 + *S. cerevisiae*	C	LR95C
*Lacticaseibacillus rhamnosus* RW-9595M	M	LRWM
*Lacticaseibacillus rhamnosus* RW- 9595M + *S. cerevisiae*	C	LRWC
Non-inoculated; non-fermented	NF	NFWB

**M, monoculture; C, co-culture; NF, non-fermented.**

### Monitoring Bacterial Growth and pH

Fermented samples were diluted with sterile saline (1:9 w/v) for homogenization. Viable (cultivable) lacticaseibacillus and yeast counts were determined by spread-plating 10-fold serial dilutions [in 0.1% (w/v), sterilized peptone water] on MRS-L agar (selective for *Lacticaseibacillus* due to lack of lactose consumption by yeast) or in YEPAL agar (selective for the Yeast due to lack of consumption of lactic acid by *Lacticaseibacillus*). The plates were incubated aerobically during 48 h at 37°C for lactic acid bacteria strains and 30°C for yeast, followed by counting the number of colony-forming units (CFU) per g of WB in samples taken during mono- and co-culture. Each measurement was performed in triplicate. The pH value was measured by a VWR pH meter SB70P (VWR, Pennsylvania, United States).

#### Viscosity Analysis

Viscosity of fermented substrates was measured directly with a Rheometer AR-G2 (with a standard size vanned rotor, TA Instruments, Herts, United Kingdom). Before the measurement, the samples of WB/WP fermented (20 g) were mixed with sterile water (25 g). Sample viscosity was measured at 21°C in accelerating shear rate from 2 to 500 s^–1^, followed by comparing samples at the 100 s^–1^ shear rate. The analysis was carried out in triplicate.

#### Scanning Electron Microscopy

The fermented bran samples were fixed with 2.5% glutaraldehyde buffered in sodium cacodylate 0.1 M, pH 7.3, and washed three times with buffer. Dehydration of the samples was carried out with ethanol from 30% (v/v) to 50% (v/v) to 70% (v/v) to 95% (v/v) and absolute, 3 min × 15 min then by hexamethyldisilazane (Fluka, Buchs, Switzerland). A scanning electronic microscope JEOL, JSM6360LV (Tokyo, Japan) was used to examine samples.

#### Analysis of Bioingredients and Digested Samples

At the end of the fermentation, the solid fermented medium was thermally treated (95°C for 15 min) and freeze-dried to obtain 6 different bioingredients and the control sample NFWB (Table 1). Water/salt-soluble extracts (WSE) were prepared according to the modified method described by Osborne (Weiss et al., 1993; Arte et al., 2015). An aliquot of the NFWB was diluted (1:10) with Tris-HCl 50 mM (pH 8.8), stirred for 1 h at room temperature, then centrifuged at 20,000 × *g* for 20 min. The water/salt-soluble fraction in the supernatants were stored at −20°C for further analyses.

#### *In vitro* Simulation of Digestion

The method described by Minekus et al. (2014) with some modifications was used for *in vitro* gastrointestinal digestion of bioingredients and NFWB. The three stages of digestion were mouth, gastric and small intestine. The composition of the salivary (SSF), gastric (SSG) and intestinal (SSI) juices were as described previously (Minekus et al., 2014). A saliva solution containing 1,500 U/mL α-amylase (Sigma, Germany) and diluted in 0.3 M CaCl_2_ was used to simulated mastication. The simulated saliva 1.4 mL (75 U/mL in the final mixture) was added to 2.5 g of solid and mixed thoroughly to obtain a paste like-consistency with 10 mL of SSF electrolyte stock solution, 71.4 μL of CaCl_2_ and 2.78 mL of water for 2 min at 37°C. In gastric conditions, 10.5 mL of SSG electrolyte stock solution, 7 μL of CaCl_2_, 2.24 mL pepsin (2,000 U/mL in the final digestion mixture, Sigma) and 0.973 mL of water were added to the remaining fluid. Afterward, the pH was decreased until 3 using 6 M HCl and incubated for 2 h at 37°C and 80 rpm. For small intestine conditions, 15.4 mL of SSI electrolyte stock solution, 7 mL pancreatin from porcine pancreas (100 U/mL final concentration, Sigma), 3.5 mL bile salt mixture (Sigma), 56 μL of CaCl_2_ and 1.8 mL of water were added to the remainder of the mixture. The pH was increased to 7 with 4 M NaOH, continuing incubation for 2 h at 37°C and 80 rpm. The sample solution was then placed in 6–8 kDa molecular weight cut-off dialysis tubing and dialysed 24 h to remove low molecular mass digestion products. At the end, the digestion mixtures were lyophilized to obtain the digested samples LR11M to NFWB (Table 1).

### Chemical Analysis

Samples were dried in an oven at 130°C for 5 h to determine moisture content [American Association of Cereal Chemists (AACC), 1975]. The Kjeldahl method was used to measure organic nitrogen concentration [American Association of Cereal Chemists (AACC), 1976]. Proteolysis was determined by measuring the concentration of proteins, peptides and free amino acids in the water/salt-soluble extracts (WSE) (Weiss et al., 1993; Arte et al., 2015). The concentration of soluble proteins and peptides were determined by the Bradford and the *o*-phthaldialdehyde (OPA) method, respectively (Bradford, 1976; Church et al., 1985). Quantitative analysis of free amino acids was performed using the EZ:faast kit obtained from Phenomenex (Torrance, CA, United States). After solid phase extraction, derivatization and liquid/liquid extraction of the derivatized amino acids, UHPLC-MS/MS was used to analyze the derivatized samples.

Sugars (lactose, glucose, and galactose), organic acids (lactic acid and acid acetic) and ethanol in all the samples were determined using HPLC following the same equipment and conditions reported previously (Bertsch et al., 2019). Before injection (15 μL), triplicate samples were diluted two-thirds with Milli-Q water, centrifuged at 20,000 × *g* for 15 min at 4°C, and filtered (pore size of 0.45 μm; Chromspec Syringe Filter; Chromatographic Specialties, Brockville, ON, Canada). Acetate, lactate (all from Sigma-Aldrich) and ethanol (JT Baker Chemical) were combined for use as an external standard for quantification. Assays from Megazyme International (Wicklow, Ireland) were used to determine the contents of xylose (K-XYLOSE), starch (K-TSTA-50A/K-TSTA-100A), β-glucan (K-EBHLG) and insoluble and soluble dietary fiber (K-RINTDF) from bioingredients or non-fermented wheat bran. The reducing sugar content was estimated with the dinitrosalicylic acid method (Miller, 1959).

Index of solubilisation of dietary fiber: IDF/SDF = Content of insoluble dietary fiber/content of soluble dietary fiber.

#### Determination of Water-Soluble Polysaccharides (WSP)

Ethanol precipitation was used for isolation and purification of water soluble polysaccharides (WSP) as described previously (Dubois et al., 1951; Miller, 1959; Tieking and Gänzle, 2005; Leroy and De Vuyst, 2016) with some modifications. Samples of bio-ingredients (0.5 g) were mixed with deionised water at 500 rpm × 1 h at room temperature. Then the solids from WB and cells were separated by centrifugation at 12,000 × *g* for 15 min at 4°C. The WSP were precipitated from the supernatant with 3 volumes 95% ethanol at 4°C for 24 h, and collected by centrifugation at 12,000 × *g* for 20 min. After dissolving the pellets in deionized water, the sample was freeze-dried. The lyophilized samples were treated with pancreatic amylase and amyloglucosidase 37°C for 4 h, then the pH was adjusted to 8.2 and heated to 95°C × 15 min (K-RINTDF Assay Kit). The next step was the protein hydrolysis with a protease at 60°C for 30 min (K-RINTDF Assay Kit). The pH was adjusted to 4.5, followed by two rinses of the precipitate with ethanol. After dialysis (3,500 Da MWCO, Fisher) for 3 days, with two changes of water per day, the WSP solution was freeze-dried. At the end, the total sugars were determined by the phenol/sulfuric acid method (Dubois et al., 1951) with glucose as a standard, and the results are expressed in g glucose per kg of sample. The NFWB was used as control.

#### Extraction and Separation of Free and Bound Fractions of Phenolic Compounds

Free and bound fractions of phenolic compounds were extracted as described previously (Singleton et al., 1999; Prückler et al., 2015) with the following changes: to obtain the free (non-bound) phenolic acid fraction, 50 mg DM was treated twice with 5 mL 80% methanol (Roth, HPLC grade). The extract was centrifuged, the final volume was measured, filtered (pore size of 0.45 μm) and used to determine free phenolic acid. To obtain the fraction of bound phenolic compounds, the residues of the above extraction were hydrolyzed with 1.5 mL of 2 M NaOH at 25°C for 4 h with shaking. The solution was then brought to pH 2 with 6 M HCl, extracted with ethyl acetate and evaporated to dryness under continuous nitrogen gas flush. Finally, after dissolving in 10 ml methanol, centrifugation at 10,000 × *g* for 10 min, the supernatant was stored at −20°C.

#### Determination of Total Phenolic Content (TPC)

Total phenolic content was determined in free and bound fractions using the procedure described previously (Singleton et al., 1999) with slight modifications. Briefly, the extracts (20 μL) were added to 100 μL Folin-Ciocalteu (Sigma-Aldrich F9252) reagent for 8 min, then the reaction was neutralized with 80 μl 7.5% Na_2_CO_3_, followed by 60 min incubation, then measuring absorbance at 765 nm. A standard curve of gallic acid was prepared and total phenolic content expressed as milligrams of gallic acid equivalents per gram dry weight sample (mg GAL/g DW). The percentage of recovery (unabsorbed) of TPC in DS = (amount of TPC in DS after digestion/Amount of TPC before digestion)^∗^100.

Total TPC = TPC of Free + TPC of bound fraction.

#### Oxygen Radical Scavenging Capacity (ORAC) Assay

The sample (extract from free or bound fractions of phenolic compounds) was diluted with 0.075 M phosphate buffer (pH 7.0) in black 96-well plates (Corning Scientific, Corning, NY, United States). The reaction mixture contained 20 μL of the sample or of Trolox standard and 200 μL of fluorescein (Sigma F6377). After incubation at 37°C for 20 min in a plate reader, 75 μL of (8.6 mg/mL) AAPH solution was quickly added to each well. A Fluostar Galaxy was used to measure fluorescence intensity at 485 nm for excitation and 520 nm for emission for 35 cycles every 210 s. The ORAC value was expressed as micromole Trolox equivalents per gram DM (μmol TE/100 g DM).

The percentage of antioxidant activity recovered in DS = (ORAC value in DS after digestion/ORAC value before digestion)^∗^100.

Total ORAC value = ORAC value of Free + ORAC value of bound form in samples.

#### Profiling of Phenolic Acids

A Waters Acquity Ultra-Performance TM LC system (Waters), equipped with a quaternary pump system (Waters) was used for UPLC analysis, with an Acquity high-strength silica (HSS) T3 column (150 mm × 2.1 mm internal diameter, 1.8 mm particle size, Waters) containing 100% silica particles as the stationary phase. The separation of the phenolic compounds was carried out following the same conditions reported previously (Bertsch et al., 2019). A TQD mass spectrometer (Waters) equipped with a Z-spray electrospray interface was used for mass spectrometry in negative mode and multiple reaction monitoring (MRM) was used to acquire data with Mass Lynx 4.1 software.

% Bioaccessibility = (Amount of free phenolic acid before digestion - amount of free phenolic acid in DS after digestion/Amount of phenolic acid before digestion) × 100.

The percentage of total phenolic acids recovered (unabsorbed) in DS = (amount of total phenolic acid (TPC) in DS after digestion/Amount of total phenolic acid before digestion) × 100.

The percentage of bound phenolic acids recovered (unabsorbed) in DS = (amount of bound phenolic acid in DS after digestion/Amount of total phenolic acid before digestion) × 100.

Total phenolic acids = Sum of free phenolic acid + Sum of Bound phenolic acid of the samples.

Free phenolic acids = Sum of free phenolic acid of the samples (Singleton et al., 1999; Dall’Asta et al., 2016; Swieca et al., 2017).

### Statistical Analyses

Results were expressed as the means of three triplicate analyses and three independent fermentations were conducted. One-way ANOVA was carried out with a Tukey adjustment to test for significance (*P* > 0.05) among the replicates using GraphPad Prism 6 (GraphPad software). Biochemical properties of the bioingredients were analyzed through discriminant analysis (DA), using the software XLSTAT-Premium version 2017 (Addinsoft). Correlations between variables were measured using the Pearson correlation test (*r*).

## Results

### Growth and Metabolite Production During Fermentation of WB/WP

**FIGURE 1:**
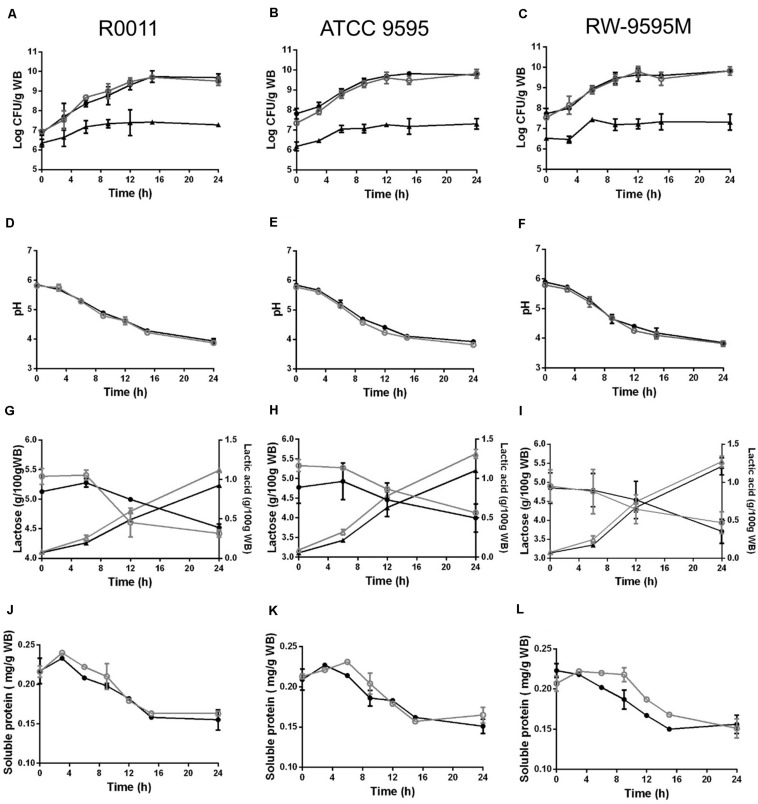
Growth **(A–C)**, pH **(D–F)**, lactose consumption (∘,•); lactic acid production (Δ, ▲) **(G–I)**, and soluble protein **(J–L)** change during fermentation by *Lacticaseibacillus rhamnosus* R0011 in **(A,D,G,J)**; ATCC 9595 in **(B,E,H,K)** and RW-9595M in **(C,F,I L)** in mono (black) or co-culture (gray) with the yeast *S. cerevisiae*.

During fermentation, the strains of lactic acid bacteria reached cell densities ranging from 6.5 (at 0 h) to 9.8 log CFU/g (at 24 h) and from 6.3 to 7.3 log CFU/g for the yeast (Figures 1A–C). No differences in growth were observed between mono and co-culture for all strains. The pH decreased from 5.7 to 3.8 at the end of the culture (Figures 1D–F). The lactose from whey permeate was the major carbon source consumed by the LAB strains and consequently lactic acid was produced (Figures 1G–I). No significant differences were observed between strains or type of culture (mono or co-culture) in lactose consumption during fermentation (*P* > 0.05). The amount of lactic acid produced by strains ATCC 9595 and RW-9595M was higher (15–19%) than R0011 in both mono and co-culture (*P* < 0.05). In monoculture, the lactic acid in the medium was significantly higher (13–15%) than in co-culture for R0011 and ATCC 9595 (*P* < 0.05), respectively. In contrast, no differences were detected in lactic acid production by strain RW-9595M in mono or co-culture. Ethanol (2%) was identified after 6 h of fermentation in co-culture for all strains. Additionally, the results showed a decrease in soluble protein content of the medium in both mono and co-culture (Figures 1J–L).

### Viscosity of the Fermented WB/WP Mixtures

**FIGURE 2:**
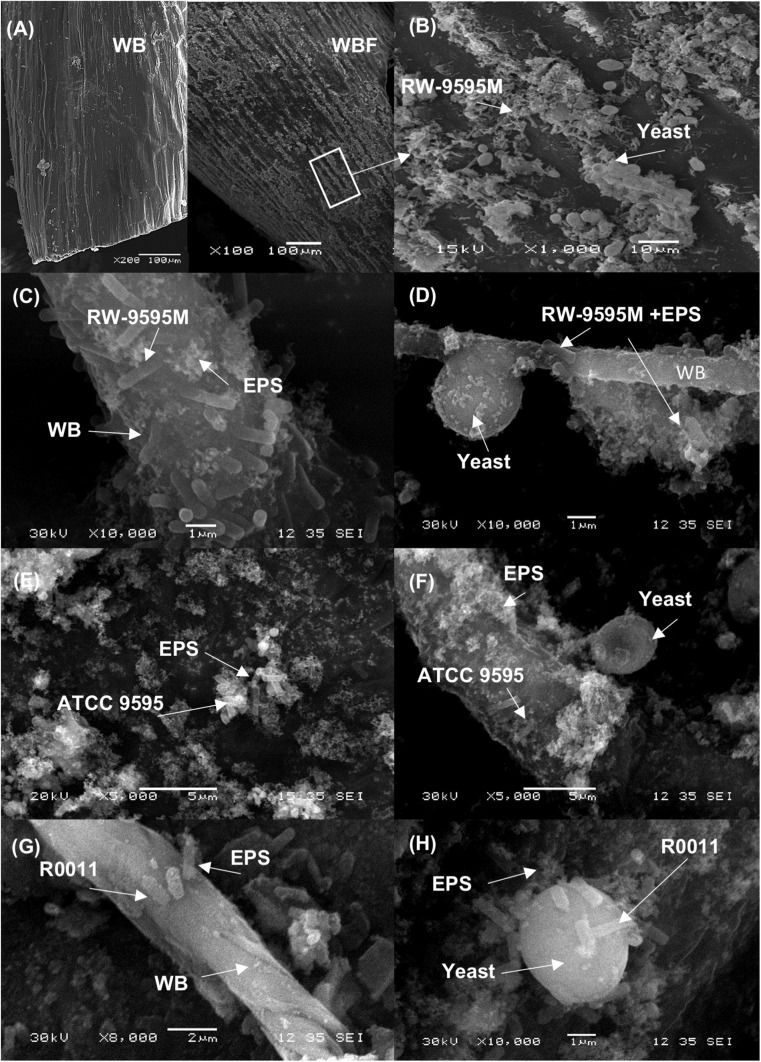
Scanning electron micrograph of wheat bran (WB) fermented 24 h using the EPS producing *L. rhamnosus* RW-9595M and *S. cerevisiae*. In panel **(A)** the surface of non-fermented wheat bran (WB) and **(B)** the surfaces of fermented wheat bran (WBF) covered by material resembling microorganisms and EPS by appearance. In the panels a higher magnification of the bacterial cells of *L. rhamnosus* RW-9595M **(C,D)**, *L. rhamnosus* ATCC 9595 **(E,F)** or *L. rhamnosus* R0011 **(G,H)**, and EPS in mono and co-culture are observed on the fermented wheat bran surface.

The six fermented WB/WP mixtures revealed a shear thinning behavior (pseudoplastic) regardless of the strain used or the fermentation method (mono or co-culture) at 24 h (Supplementary Figure S1A). However, the WB/WP fermented by RW-9595M in mono or co-culture showed a more pronounced decrease in viscosity compared to non-fermented WB/WP (Supplementary Figure S1). The results show that the pseudoplastic behavior increased over time in mono as well as in co-culture samples for the mixture fermented by RW-9595M at 0, 4, 6, 12, and 24 h (Supplementary Figures S1B,C). Bacterial growth (log CFU/g) was negatively correlated with viscosity (Pa.s) (Pearson index *r* = −0.89, *P* = 0.04, and *r* = −0.97, *P* = 0.006 for mono and co-culture, respectively) (Supplementary Figure S2). When the shear rate was 100 s^–1^, a decrease of 50% of the apparent viscosity was observed at 4 h for the monoculture or at 6 h for the co-culture (Supplementary Figure S2A). Interestingly, at this moment ropy strands were visible (Supplementary Figure S3) with a white pellicle during fermentation, which homogeneously covered the substrate in comparison to the medium fermented by R0011 or ATCC 9595. The three *L. rhamnosus* strains are known to produce different quantities of the same exopolysaccharide (EPS) associated with cell growth. At 24 h of fermentation (Figure 2), scanning electron microscopy showed that the fiber surface of fermented WB/WP was covered with a pellicle that was not present in non-fermented WB/WP (Figures 2A,B). At a higher magnification, the *L. rhamnosus* strains, yeast and exopolysaccharides are visible on the wheat bran surface (Figures 2C–H).

### Chemical Composition of Bioingredients Before and After Digestion

**FIGURE 3:**
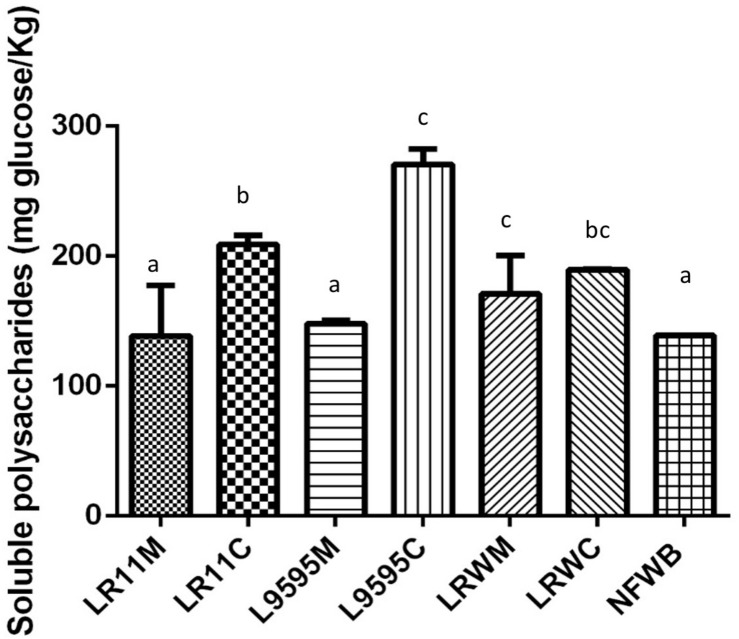
Water soluble polysaccharide (WSP) content of bioingredients obtained in mono or co-culture by the strains of *L. rhamnosus* R0011, ATCC 9595, and RW-9595M. Data with different letters in the same column are significantly different (*P* < 0.05). The data are the means of three independent experiments ± standard deviations (*n* = 3).

Dried fermented cereal-based products were obtained from a WB/WP mixture. All the WB/WP preparations, fermented or not, contained insoluble dietary fiber at 34–39% of dry matter (Table 2). Soluble dietary fiber and starch content were similar between the fermented and non-fermented (NFWB) samples (*P* > 0.05). Nevertheless, the beta-glucan content of LR95C (co-cultured ATCC 9595) was significantly (*P* < 0.05) higher (45%) than LR95M (monocultured ATCC 9595). No significant differences in beta-glucan content were found for bioingredients after mono or co-culture with R0011 (LR11M and LR11C) or monocultures of RW-9595M (LRWM) and ATCC 9595 (LR95M). In contrast, the co-cultured RW-9595M (LRWC) and ATCC 9595 (LR95C) bioingredients had similar beta-glucan content to NFWB. Moreover, the content of water-soluble polysaccharides (WSP) of the WB/WP mixtures increased significantly (*P* < 0.05) throughout the co-culture. The presence of yeast improved the content of WSP of WB/WP by 49%; 33.5 and 26.4% for the strains ATCC 9595, R0011 and RW-9595M, respectively (Figure 3). In co-culture, the WSP content was significantly higher (*P* < 0.05) than monoculture by 31% for R0011; 45.5% for ATCC 9595 and 9% for RW-9595M, respectively.

**TABLE 2:** Chemical composition (%DM) of bioingredients and control non-fermented wheat bran (NFWB).

Component	LR11M	LR11C	LR95M	LR95C	LRWM	LRWC	NFWB
Dry matter (%)	94.8 ± 0.4	94.7 ± 0.4	94.9 ± 0.2	95.0 ± 0.1	94.1 ± 0.1	95 ± 0.3	94.5 ± 0.1
Ash (%)	5.9 ± 0.1	5.5 ± 0.1	5.8 ± 0.3	5.4 ± 0.8	6.2 ± 0.3	5.9 ± 0.2	5.8 ± 0.1
Protein (%)	13.9 ± 0.1	14.3 ± 0.1	13.9 ± 0.3	14.1 ± 0.2	13.8 ± 0.4	14.5 ± 0.5	14.4 ± 0.3
IDF (%)	37.4 ± 2.9	38.0 ± 1.1	34.3 ± 0.8	36.7 ± 1.5	35.7 ± 1.2	39 ± 0.9	38.6 ± 0.6
SDF (%)	3.9 ± 0.3	3.7 ± 0.0	3.5 ± 0.1	2.8 ± 0.3	2.7 ± 0.2	3.2 ± 0.3	3.1 ± 0.6
Index SDF/IDF	0.104	0.097	0.102	0.076	0.075	0.08	0.08
Starch (%)	8.2 ± 0.2	8.7 ± 0.3	9.7 ± 0.3	8.6 ± 0.6	8.3 ± 0.1	8.5 ± 0.2	7.6 ± 1.9
BGluc (%)	2.9 ± 0.4^*a*^	2.7 ± 0.0^*a*^	2.3 ± 0.3^*a*^	4.2 ± 0.0^*b*^	3.2 ± 0.1^*ab*^	3.9 ± 0.1^*ab*^	3.6 ± 0.1^*ab*^
Sol.protein (mg/g)	1.3 ± 0.1^*a*^	1.4 ± 0.0^*a*^	1.5 ± 0.0^*a*^	1.6 ± 0.1^*a*^	1.3 ± 0.05^*a*^	1.4 ± 0.0^*a*^	5.6 ± 0.3^*b*^
Peptides (mg/g)	60.3 ± 3.7	40.9 ± 0.8	44.2 ± 2.6	63.1 ± 5.1	55.9 ± 17.1	53.3 ± 5.3	57.1 ± 4.4
Xylose (mg/g)	20.9 ± 1.7^*a*^	17.2 ± 2.4^*a*^	20.1 ± 0.6^*a*^	26.2 ± 0.5^*a*^	18.0 ± 0.5^*a*^	23.1 ± 1.4^*a*^	34.1 ± 1.1^*b*^
Lactose (mg/g)	138.9 ± 11^*a*^	155.1 ± 6.8^*a*^	152.5 ± 7.6^*a*^	153.4 ± 17.8^*a*^	157.7 ± 22.3^*a*^	156.4 ± 1.7^*a*^	235 ± 0.0^*b*^
Galactose (mg/g)	1.7 ± 0.1^*a*^	nd*	1.6 ± 0^*a*^	0.4 ± 0.0^*b*^	1.5 ± 0.2^*a*^	nd*^b^	3.4 ± 0.6^*c*^
Lactic acid (mg/g)	44.7 ± 2.8^*a*^	40.0 ± 0.4^*a*^	58.6 ± 3.7^*b*^	52.4 ± 4.8^*b*^	61.5 ± 7.8^*b*^	53.7 ± 1.1^*b*^	3.5 ± 0.6^*c*^

**The data are the means of three independent experiments ± standard deviations (n = 3). Data with different letters in the same column are significantly different (P < 0.05) ^∗^nd, not detected; IDF, insoluble fiber; SDF, soluble dietary fiber; Sol.protein, soluble protein; BGluc, beta glucans*.*

Galactose, lactose and xylose contents were higher in NFWB by 56–100, 33–41, and 24–50% in comparison to the fermented bio-products (*P* < 0.05), respectively (Table 2). Lactic acid was the major fermentation end-product and was higher (91–95%) in the bioingredients than in NFWB (*P* < 0.01). Total protein content did not significantly differ between the bioingredients and the non-fermented substrate (*P* > 0.05). Conversely, significant differences (*P* < 0.05) were observed in the fraction of soluble protein. Likewise, in culture medium, the amount of soluble protein was higher (72%) in the NFWB than in fermented WB/WP. The peptide content was similar between the non-fermented and fermented samples.

In this study, the total free amino acid content was higher in the bioingredients (23.5–43%) compared to NFWB (*P* < 0.05) (Table 2). A heat map was constructed to visualize the result of hierarchical clustering of the profile data and to identify changes in free amino acid content among bio-products (Supplementary Figure S4). The dendrogram of the heat map shows groups of NFWB (non-fermented) and the bioingredients (fermented). Thus, in fermented bioproducts, 63% of the amino acids were significantly higher (*P* < 0.05) after bioprocessing (Arg, Gln, Ser, Gly, Thr, Ala, GABA, Pro, Asp, His, Glu, and Phe) in comparison to NFWB.

The concentration of GABA, a functional amino acid derivative, was slightly higher (between 26 and 47%) as a consequence of fermentation compared to non-fermented wheat bran (NFWB). Furthermore, the heat map shows that bioproducts LR11M (mono) and LR11C (co-culture) from *L. rhamnosus* R0011 were clustered together. Likewise, the analysis distinguished the amino acid profiles between the bioingredients from monoculture (LR95M and LRWM) or co-culture (LR95C and LRWC) of *L. rhamnosus* ATCC 9595 and RW-9595M. For strains ATCC 9595 and RW-9595M, the bioingredient obtained from co-culture showed an amino acid profile enriched in Lys, Val, Leu, Phe, Ile, and Glu.

Samples were hydrolyzed through simulated buccal, gastric and intestinal stages. The term digested sample (DS) refers to a portion (with a solubility ranging between 50 and 63%) which remained after the *in vitro* assay and would be degraded and converted in the colon by the intestinal microbiota. Dietary fiber was the major component of the digested samples, with values ranging from 54.3 to 58.5% (Table 3). The soluble dietary fiber was similar among the samples and ranged from 8.1 to 10.7%.

**TABLE 3:** Chemical composition (%DM) of digested bioingredients and non-fermented mixture of wheat bran and whey permeate (NFWB).

Component	LR11M	LR11C	LR95M	LR95C	LRWM	LRWC	NFWB
Dry matter (%)	94.7 ± 0.3	95.3 ± 0.5	95.3 ± 0.6	95.4 ± 0.1	95.0 ± 0.1	95.0 ± 0.3	82.1 ± 2.6
Protein (%)	14.5 ± 0.3^*a*^	15.6 ± 0.1^*b*^	15.6 ± 0.0^*b*^	14.9 ± 0.1^*b*^	15.2 ± 0.1^*b*^	15.1 ± 0.0^*b*^	14.2 ± 0.0^*a*^
IDF (%)	55.1 ± 0.3	54.3 ± 1.2	55.4 ± 0.8	58.5 ± 1.3	56.3 ± 0.1	57.7 ± 0.1	56.8 ± 0.5
SDF (%)	8.1 ± 1.1	10.4 ± 0.6	10.7 ± 0.5	9.3 ± 1.6	10.0 ± 1.1	9.4 ± 1.1	8.2 ± 0.1
Index ISF/IDF	0.145	0.19	0.19	0.154	0.175	0.161	0.145
Soluble protein (mg/g)	6.1 ± 0.4^*a*^	7.3 ± 0.1^*a*^	8.2 ± 0.05^*b*^	7.4 ± 0.7^*a*^	9.7 ± 0.07^*b*^	8.7 ± 0.2^*b*^	9.0 ± 0.1^*b*^
Peptides (mg/g)	84.7 ± 3.2	95.2 ± 1.9	87.4 ± 1.3	89.6 ± 3.1	94.1 ± 2.3	83.9 ± 2.3	83.9 ± 4.4
Xylose (mg/g)	75.2 ± 14^*a*^	46.0 ± 4.8^*a*^	98.6 ± 1.7^*b*^	83.6 ± 1.6^*b*^	80.7 ± 0.0^*b*^	101.7 ± 0.8^*b*^	107 ± 0.2^*b*^
Lactose (mg/g)	22.4 ± 0.2	23.0 ± 0.4	22.9 ± 0.6	21.2 ± 0.4	23.5 ± 0.3	25.4 ± 1.6	23.4 ± 0.0
Glucose (mg/g)	1.3 ± 0.0^*a*^	1.2 ± 0.0^*ab*^	1.5 ± 0.0^*c*^	1.1 ± 0.1^*b*^	1.4 ± 0.03^*c*^	1.3 ± 0.1^*a*^	1.5 ± 0.1^*a*^
Lactic acid (mg/g)	6.4 ± 0.0^*a*^	5.0 ± 0.0^*b*^	7.6 ± 0.1^*c*^	6.6 ± 0.2^*a*^	7.9 ± 0.1^*c*^	7.5 ± 0.5^*c*^	0.4 ± 0.5^*d*^

**Data with different letters in the same column are significantly different (P < 0.05). IDF, insoluble fiber; SDF, soluble dietary fiber. The data are the means of three independent experiments ± standard deviations (n = 3).**

The protein content of the co-cultured bioingredients LR11C to LRWC was higher (9%) than digested NFWB and LR11M (*P* < 0.01). The soluble protein content in digested LR11M and LR11C was lower (33 and 19%, respectively) than the digested control NFWB, LR95M and LRWC (*P* < 0.05). In contrast, the peptide content in digested samples LR11C and LRWM was higher (12 and 11%, respectively) than in digested NFWB (*P* < 0.05). No differences were found in sugar content (reducing sugar, xylose, lactose, or glucose) between the digested samples from fermented or non-fermented products (*P* > 0.05), while the lactic acid in the digested bioingredients was higher (92–95%) than the non-fermented control.

### Impact of Fermentation and *in vitro* Digestion on Bioactive Compounds and Antioxidant Activity

#### Total Phenolic and Antioxidant Activity

In general, the bound TPC and antioxidant activity of bioingredients and digested samples were higher than the free fraction (Table 4). The free fraction represented 3–32% of TPC and 29–45% of the antioxidant activity in bioingredients. Free TPC and antioxidant activity of the LRWM bio-product LRWM were similar to NFWB (*P* > 0.05) and higher than the other bioingredients. Nevertheless, free TPC content did not differ significantly between monoculture and co-culture for the bioingredients fermented using RW-9595M (LRWM-LRWC) or ATCC 9595 (LR95M-LR95C).

**TABLE 4:** Total phenolic content (TPC) and antioxidant activity (ORAC) of bioingredients **(A)** and the digested samples **(B)**.

(A) Bioingredients before digestion.							
Component	LR11M	LR11C	LR95M	LR95C	LRWM	LRWC	NFWB

**Total phenolic content (mg/100 g DM)**							
Free	7 ± 1.6^*a*^	22 ± 3.7^*b*^	27 ± 0.6^*b*^	33 ± 3.3^*b*^	52 ± 2.6^*cd*^	48 ± 4.5^*c*^	62 ± 5.1^*d*^
Bound	231 ± 14.4^*a*^	246 ± 38.2^*a*^	308 ± 4.3^*b*^	237 ± 7.5^*a*^	437 ± 0.62^*c*^	265 ± 0.37^*a*^	132 ± 3.0^*d*^
Total	238 ± 12.8^*a*^	269 ± 34.4^*a*^	336 ± 3.7^*b*^	271 ± 4.2^*ab*^	489 ± 1.9^*c*^	313 ± 4.2^*b*^	194 ± 2.1^*d*^
**ORAC (μmol Trolox/100 g DM)**							
Free	5,306 ± 6^*a*^	6,668 ± 588^*b*^	6,020 ± 23^*ab*^	6,811 ± 118^*ab*^	8,642 ± 275^*c*^	7,644 ± 575^*a*^b	7,990 ± 134^*c*^
Bounded	12,338 ± 294^*a*^	12,925 ± 99^*a*^	15,011 ± 400^*a*^	14,686 ± 326^*a*^	21,313 ± 806^*b*^	16,876 ± 867^*a*^	9,859 ± 86^*c*^
Total	17,644 ± 288^*a*^	19,593 ± 687^*a*^	21,031 ± 377^*a*^	21,497 ± 444^*a*^	29,954 ± 1081^*b*^	24,520 ± 1442^*c*^	17,849 ± 49^*a*^

**(B) Bioingredients after digestion**							

**Component**	**LR11M**	**LR11C**	**LR95M**	**LR95C**	**LRWM**	**LRWC**	**NFWB**
**Total phenolic content (mg/100 g DM)**							
Free	90 ± 1.6^*a*^	94 ± 1.7^*a*^	90 ± 0.1^*a*^	98 ± 4.5^*a*^	99 ± 1.1^*a*^	108 ± 3.5^*ab*^	116 ± 4.2^*b*^
Bound	413 ± 27.3^*a*^	589 ± 1.9^*b*^	391 ± 0.4^*a*^	559 ± 2.2^*bc*^	527.6 ± 11^*bc*^	429 ± 10.2^*a*^	540 ± 5.1^*c*^
Total	503 ± 26^*a*^	683 ± 3.5^*b*^	481 ± 0.5^*a*^	656.1 ± 6.7^*b*^	626.8 ± 12^*bc*^	537 ± 6.7^*a*^	656 ± 0.9^*b*^
**ORAC (μmol trolox/100 g DM)**							
Free	10,656 ± 494^*a*^	11,241 ± 209^*ab*^	10,469 ± 1,636^*ab*^	11,903 ± 396^*c*^	8,758 ± 32^*b*^	9,822 ± 140^*b*^	10,413 ± 333^*a*^
Bound	16,203 ± 611^*a*^	17,028 ± 290^*a*^	15,036 ± 1,348^*a*^	22,244 ± 777^*b*^	24,287 ± 323^*b*^	23,275 ± 502^*b*^	23,451 ± 554^*b*^
Total	26,859 ± 1,105^*a*^	28,269 ± 81.3^*a*^	25,505 ± 984^*a*^	34,148 ± 381^*b*^	3,3045 ± 355^*b*^	33,098 ± 642^*b*^	33,866 ± 221^*b*^

**Data with differents letters in the same column are significantly different (P < 0.05). The data are the means of three independent experiments ± standard deviations (n = 3)*.*

The amount of bound and total (sum of free and bound fractions) TPC as well as the antioxidant activity in all the bio-products (Table 4) were higher in fermented bioingredients than in NFWB (*P* < 0.001). The bioingredient fermented by RW-9595M in monoculture (LRWM) showed the maximum increase in TPC (bound and total), which was higher by 69.8 and 60% compared to NFWB. The bound and total TPC content was higher in monoculture in comparison to co-culture for RW-9595M (39 and 30% for bound and total phenolic content) and ATCC 9595 (23 and 19%) (*P* < 0.01). Likewise, the antioxidant activity of the bound fraction of LRWM was improved by 54% in comparison to NFWB. Furthermore, the antioxidant capacity of the bound and the total fractions of LRWM (monoculture) were higher (21 and 18%, respectively) than in the co-culture product LRWC (*P* < 0.01). Mono and co-cultured products fermented with R0011 (LR11M-LR11C) or ATCC 9595 (LR95M-LR95C) (*P* > 0.05) did not differ significantly. These results show a positive correlation between free and bound TPC and antioxidant activity (ORAC) of bioingredients (Pearson index *r* = 0.86 and *r* = 0.92, *P* < 0.0001, respectively).

After digestion, the free fraction of TPC represented 14–21% of TPC and 59–73% of the antioxidant activity (Table 4). The free TPC of LRWC (co-culture of RW-9595M) was similar to NFWB and it was the highest among the fermented samples. For strains R0011 and ATCC 9595, no differences in free TPC were observed between mono and co-culture. The antioxidant activity of the free fraction was similar in digested samples whatever their origin. On the other hand, the content of bound and total TPC in digested WB/WP after co-culture with R0011 and ATCC 9595 (LR11C and LR95C) and monoculture RW-9595M (LRWM) was similar to digested NFWB and higher than the other samples. The antioxidant activity of bound and total fractions revealed one group composed of digested samples from yeast co-culture with either ATCC 9595 (LR95C) or RW-9595M (LRWM-LRWC), which were similar to digested NFWB and higher than the second group containing the digested samples fermented with strain R0011 (LR11M-LR11C) and yeast co-culture of ATCC 9595 (LR95M) (*P* < 0.01).

**FIGURE 4:**
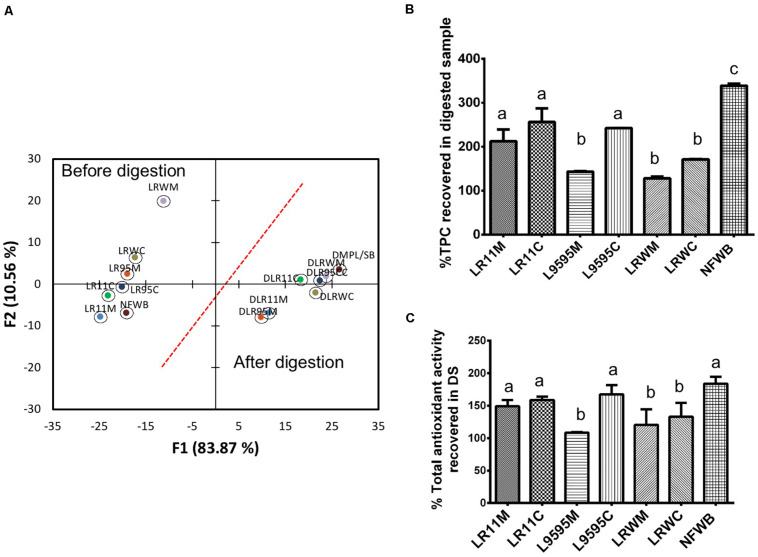
**(A)** Biplot showing the results of the discriminant analysis of total (free and bound) phenolic content (TPC) and antioxidant activity (ORAC total) of bioingredients before and after *in vitro* digestion. The **(B)** Comparison of the percentage of TPC and **(C)** antioxidant activity recovered (unabsorbed) in the bioingredients and non-fermented sample (NFWB). Data with different letters in the same column are significantly different (*P* < 0.05). The data are the means of three independent experiments ± standard deviations (*n* = 3).

Data collected from the free, bound and total TPC after and before the *in vitro* digestion were subjected to discriminant analysis (Figure 4A). Discriminant analysis was used to classify the data into groups according to discrete (categorical) variables. Two principal factors explained more than 92% of the variance of the independent variables. The F1 principal factor shows the effect of digestion. LRWM (monoculture of RW-9595M) is well separated from the other bioingredients and NFWB (left side of the figure). Interestingly, after digestion, the properties of the digested bioingredients and NFWB were more similar.

In spite of the similarity between fermented and non-fermented digested samples mentioned above, the recovery of unabsorbed TPC in digested NFWB was higher (23–61%) than that of the digested fermented bioingredients (Figure 4B). Monoculture resulted in a lower percentage of TPC and antioxidant activity than co-culture. LRWM-LRWC and LR95M had the lowest percentage of TPC recovered. Nevertheless, higher antioxidant activity (ORAC) kept the digested NFWB superior (34 and 40%) to those of LRWM and LR95M, respectively. This was in accordance with the lower correlation detected between TPC and antioxidant activity (Pearson *r* = 0.564 *P* < 0.05) in the digested samples.

#### Phenolic Acid Profile and Bioaccessibility

Gallic acid, protocatechuic acid, ferulic acid and caffeic acid content of bioingredients differed significantly from NFWB (*P* < 0.01) (Table 5). Total free phenolic acid content was higher for LR95M-LR95C (53–59%), LRWM-LRWC (45–46%), and LR11M-LR11C (29–39%) compared to non-fermented NFWB. There was no variation in 4-hydroxybenzoic acid and shikimic acid content between the bio-products and the control. The gallic acid content in LR11M, LR95C, LRWM, and LRWC was similar to NFWB and lower than that of LR95M. The protocatechuic acid content was lower by 14 and 47% in bioingredients compared to NFWB.

**TABLE 5:** Profile of free and bound phenolic acids of **(A)** bioingredients and **(B)** digested samples.

Bio-ingredients	LR11M	LR11C	LR95M	LR95C	LRWM	LRWC	NFWB
**(A) Bioingredients before digestion**							
**Free acid phenolic (μg/100g DM)**							
Gallic acid	76 ± 10^*a*^	59 ± 26^*a*^	167 ± 13^*b*^	147 ± 4.8^*b*^	126 ± 19^*c*^	81 ± 11^*a*^	106 ± 0.3^*a*^
4-Hyd acid*	288 ± 34^*a*^	463 ± 57^*b*^	283 ± 53^*b*^	336 ± 47^*b*^	245 ± 92^*a*^	336 ± 7.0^*b*^	201 ± 3.0^*a*^
Caffeic acid	4,662 ± 285^*a*^	4,265 ± 224^*a*^	8,053 ± 35^*b*^	8,359 ± 931^*b*^	6,292 ± 487^*c*^	6,579 ± 738^*c*^	142 ± 11^*d*^
Ferulic acid	1,414 ± 108^*a*^	852 ± 53^*b*^	262 ± 37^*c*^	380 ± 53^*d*^	423 ± 15^*d*^	296 ± 28^*d*^	2,547 ± 10^*e*^
Protocatechuic acid	1,210 ± 294^*a*^	876 ± 91^*a*^	1,201 ± 17^*a*^	2,064 ± 199^*c*^	1,428 ± 117^*a*^	1,363 ± 74^*a*^	1,660 ± 468^*a*^
Shikimic acid	20 ± 3.9^*a*^	28 ± 2.5^*b*^	21 ± 0.6^*b*^	26 ± 4.3^*b*^	14 ± 0.3^*a*^	19 ± 3.6^*a*^	19 ± 3^*a*^
*p-*Coumaric	nd	nd	nd	nd	nd	359 ± 46	nd
Total	7,671 ± 310	6,545 ± 361	9,988 ± 78	11,311 ± 864	8530 ± 553	8,676 ± 828	4,677 ± 1215
**Bound acid phenolic (mg/100 g DM)**							
4-Hyd acid*	5 ± 0.02^*a*^	6 ± 0.22^*a*^	7 ± 0.18^*b*^	8 ± 0.40^*c*^	6 ± 0.05^*b*^	8 ± 0.53^*c*^	7 ± 0.30^*b*^
*p-*Coumaric acid	10 ± 1.25^*a*^	16 ± 0.28^*b*^	19 ± 2.3^*b*^	20 ± 0.30^*b*^	15 ± 0.21^*b*^	24 ± 0.50^*c*^	19 ± 0.18^*b*^
Caffeic acid	0.2 ± 0.03^*a*^	0.4 ± 0.02^*a*^	nd	nd	nd	0.74 ± 0.04^*b*^	nd
Ferulic acid	116 ± 7.8^*a*^	174 ± 6.9^*b*^	203 ± 2.1^*b*^	166 ± 5.45^*b*^	148 ± 7.5^*b*^	221 ± 9.1^*c*^	162 ± 5.5^*b*^
IsoFerulic acid	39 ± 1.5^*a*^	68 ± 3.2^*b*^	55 ± 5.9^*b*^	53 ± 0.2^*b*^	50 ± 5.3^*b*^	102 ± 4.7^*c*^	60 ± 2.3^*b*^
Protocatechuic acid	nd	113 ± 16^*a*^	nd	nd	nd	218 ± 70^*b*^	nd
Total	170.2 ± 10.6	264.2 ± 9.9	285.5 ± 13.1	246.6 ± 6.4	219.5 ± 13.1	356.4 ± 13.9	248.4 ± 8.3
**(B) Bioingredients after digestion**							
**Free acid phenolic (μg/100g DM)**							
Gallic Acid	31 ± 0.1^*a*^	15 ± 0.1^*b*^	15.1 ± 0.9^*b*^	23.9 ± 0.9^*a*^	22.1 ± 0.9^*a*^	9.3 ± 0.2^*c*^	17 ± 0.3^*b*^
4-Hyd acid*	91 ± 8^*a*^	145 ± 0.1^*a*^	33 ± 0.1^*b*^	78 ± 1.1^*b*^	69 ± 4.1^*b*^	47 ± 1^*b*^	223 ± 38^*c*^
Caffeic acid	687 ± 151^*a*^	1,025 ± 140^*a*^	2,113 ± 343^*b*^	2,014 ± 156^*b*^	1,299 ± 166^*ab*^	1,370 ± 317^*ab*^	202 ± 0.1^*a*^
Ferulic acid	589 ± 127^*a*^	383 ± 65^*a*^	65 ± 27^*b*^	138 ± 15^*c*^	285 ± 67^*c*^	274 ± 27^*c*^	1,210 ± 268^*d*^
Protocatechuic acid	550 ± 64^*a*^	205 ± 9^*b*^	193 ± 126^*b*^	399 ± 139^*b*^	308 ± 92^*b*^	1,049 ± 8^*c*^	1,043 ± 4^*c*^
Shikimic acid	4.4 ± 0.3^*a*^	3 ± 0.2^*a*^	4.4 ± 0.8^*a*^	4 ± 0.2^*a*^	5 ± 0.1^*a*^	6 ± 2.2^*a*^	4 ± 0.2^*a*^
Total	1,953 ± 96	1,778 ± 201	2,524 ± 363	2,705 ± 98	2,059 ± 134	2,757 ± 335	2,704 ± 302
**Bound phenolic acids (mg/100 g DM)**							
4-Hyd acid*	8 ± 0.4^*a*^	8 ± 0.2^*a*^	7 ± 0.6^*a*^	8 ± 0.1^*a*^	8 ± 0.4^*a*^	10 ± 0.4^*b*^	7 ± 0.5^*a*^
*p-*Coumaric acid	32 ± 0.5^*a*^	29 ± 2.8^*a*^	26 ± 2.3^*a*^	28 ± 0.5^*a*^	28 ± 2.2^*a*^	34 ± 2.5^*a*^	28 ± 0.2^*a*^
Caffeic acid	nd	0.6 ± 0.03^*b*^	0.9 ± 0.07^*b*^	1.7 ± 0.02^*c*^	1.4 ± 0.2^*c*^	nd	19 ± 0.1^*c*^
Ferulic acid	262 ± 6.9^*a*^	283 ± 10.3^*a*^	259 ± 9.8^*a*^	266 ± 0.41^*a*^	276 ± 12.78^*a*^	268 ± 12.8^*a*^	257 ± 15.5^*a*^
Isoferulic acid	96 ± 1.1^*a*^	91 ± 3.5^*a*^	78 ± 2.2^*b*^	100 ± 0.8^*a*^	102 ± 4.7^*ba*^	102 ± 4.7^*a*^	105 ± 5.4^*a*^
Protocatechuic acid	nd	0.5 ± 0.06^*a*^	0.3 ± 0.07^*a*^	0.6 ± 0.12^*b*^	0.3 ± 0.1^*a*^	nd	0.4 ± 0.01^*a*^
Total	398 ± 11.5	413 ± 13.9	371 ± 15	404 ± 2.4	417 ± 28.9	413 ± 27.8	399 ± 29.2

*The data are the means of three independent experiments ± standard deviations (n = 3).* *4 Hyd acid, 4 hydroxybenzoic acid.

The free ferulic acid content was lower by 44.5% (LR11M-LR11C) to 88% (LR95M to LRWC) when compared with NFWB regardless of the type of culture employed (mono or co-culture) (*P* < 0.01). Free *p*-coumaric acid was present in NFWB but was absent in the fermented products. Interestingly, the content of free caffeic acid increased 97–98% in bioingredients compared to NFWB (*P* < 0.001).

After gastrointestinal digestion, the total free phenolic acid content was reduced by 73.6% in bioingredients and by 40% in NFWB, with no differences between mono and co-culture (Table 5). The content of shikimic acid was similar before and after digestion (*P* > 0.05). The digested NFWB sample showed the highest concentration of 4-hydroxybenzoic acid and ferulic acid in comparison to the digested samples from fermented bioingredients (higher by 51–95%). Similar to bioingredients, the free caffeic acid content of digested LR95M-LR95C was higher by 90% than in digested NFWB, followed by LRWM-LRWC (85%) and LR11M-LR11C (70–80%) (*P* < 0.01).

**FIGURE 5:**
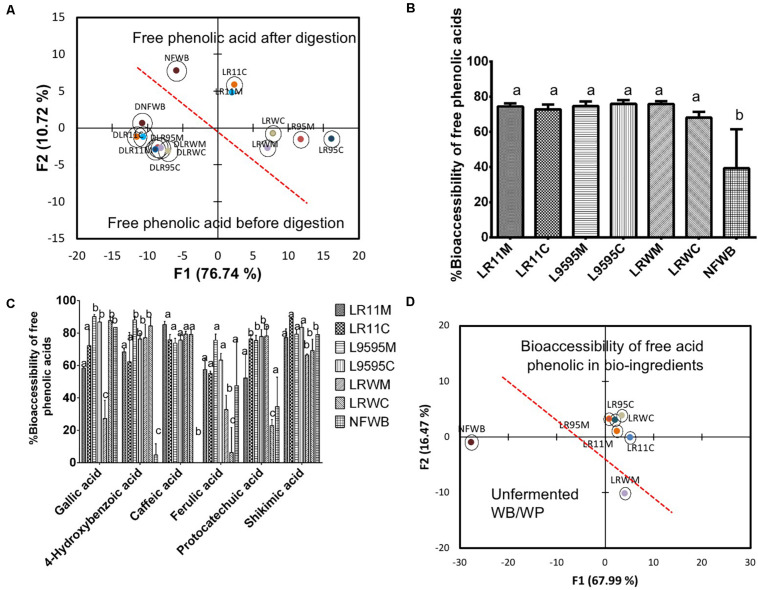
Biplot showing the results of the discriminant analysis (DA) of **(A)** free phenolic acid content of bioingredients before and after *in vitro* digestion, **(B)** % bioaccessibility of free total phenolic acids (absorbed), **(C)** % bioaccessibility of individual free phenolic acids, and **(D)** bi-plot showing the classification of the bioingredients according their free phenolic acid bioaccessibility. Data with different letters in the same column are significantly different (*P* < 0.05). The data are the means of three independent experiments ± standard deviations (*n* = 3).

Based on free phenolic acid content, discriminant analysis separated bioingredients from NFWB (Figure 5A). Two principal factors explained more than 86% of the variance. As observed previously, the bioingredients fermented by R0011 (LR11M-LR11C) were similar in mono and co-culture and different from the bioingredients fermented by strains ATCC 9595 (LR95M-LR95C) and RW-9595M (LRWM-LRWC). As was expected, digestion results in more uniformity among bioingredients and non-fermented NFWB (Figure 5A).

**FIGURE 6:**
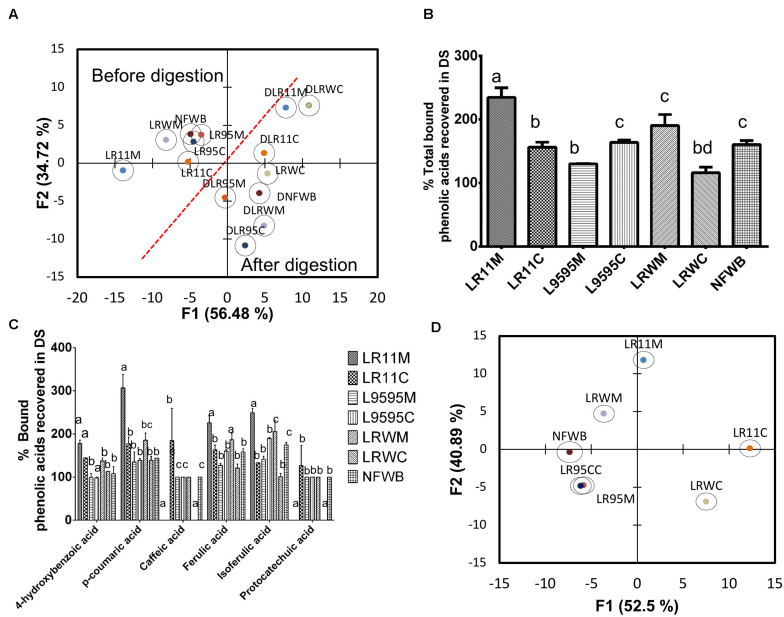
**(A)** Biplot showing the results of the discriminant analysis (DA) of bound phenolic acid content before and after *in vitro* digestion, **(B)** % of total phenolic acids recovered in digested samples of bioingredients, **(C)** % bound individual phenolic acids recovered (unabsorbed) in digested samples of bioingredient. **(D)** Biplot showing the classification of bioingredients according the % of phenolic acids recovered in digested samples by discriminant analysis. Data with different letters in the same column are significantly different (*P* < 0.05). The data are the means of three independent experiments ± standard deviations (*n* = 3).

The bioaccessibility of the free total phenolic acids was improved by 42-48% in fermented bioingredients compared to NFWB (*P* < 0.05) (Figure 5B). Bioaccessibility of free gallic and shikimic acids (in general >70%) was similar to NFWB while the bioaccessibility of caffeic and 4-hydroxybenzoic acids in bioingredients showed values superior to 60–75% (Figure 5C), which was significantly higher than NFWB (*P* < 0.01). The bioaccessibility of free protocatechuic acid was higher in LR11C through to LRWM when compared to NFWB. The bioingredients from R0011 and ATCC 9595 showed higher values (58% and more than 65%) of bioaccessible ferulic acid, respectively (Figure 5C). In contrast, both bioingredients from RW-9595M (LRWM and LRWC) showed lower ferulic acid bioaccessibility (values inferior to 35%) compared to NFWB and the other bioingredients. The discriminant analysis showed that NFWB and LRWM are different from the other bioingredients (Figure 5D). Thus, the proportion of bioaccessible phenolic acids was strain and culture dependent. The monoculture resulted in higher bioaccessibility of ferulic acid for ATCC 9595 and RW-9595M. The bioingredients contained a higher quantity of bound 4-dihydroxybenzoic, *p-*coumaric, ferulic acid and its isomer, isoferulic acid, than free forms (Figure 6A). The bioingredient from RW-9595M showed higher values of bound phenolic acid compared to the other bioingredients and NFWB.

After gastrointestinal digestion, the bound phenolic acid content increased compared to before digestion (Table 5). Discriminant analysis of bound phenolic acid content showed that digested bioingredients LRWC and LR11M could be differentiated from NFWB, except for LRWC (Figure 6A). The two principal factors explained more than 90% of the variance of variables.

The recovery of individual bound phenolic acids was superior from LLR11M (32%) compared to NFWB and the other bioingredients (Figure 6C). In contrast, compared to NFWB, the LR95M bioingredient LR95M (19% inferior) and LRWC (27% inferior) showed the lowest recovery index values (Figure 6C). For *L. rhamnosus* strains R0011 and RW-9595M, the recovery of individual bound phenolic acids was significantly higher in mono (LR11M and LRWM) than in co-culture (LR11C and LRWC) (*P* < 0.05) while no differences were found for ATCC 9595 (LR95M-LR95C) (Figures 6B,D). The LRWC bioingredient showed the lowest recovery index values for bound caffeic, isoferulic and protocatechuic acids.

## Discussion

### Growth and Viscosity During Fermentation of WB/WP

In both mono and co-culture, *L. rhamnosus* strains R0011, ATCC 9595, and RW-9595M showed similar profiles during fermentation of WB/WP medium. *L. rhamnosus* R0011 produced the lowest quantity of lactic acid in 48 h of fermentation compared to the two other strains. The presence of ethanol in co-culture was related to the metabolism of the yeast, as *S. cerevisiae* is able to convert lactic acid to pyruvate to produce ethanol and decrease the levels of lactic acid in the medium (Cheirsilp and Radchabut, 2011). The interaction of yeast with LAB during co-culture in the presence of lactose can be considered as mutualism (Ponomarova et al., 2017).

The rheological behavior in terms of viscosity of WB/WP fermented by R0011 or ATCC 9595 was not different from that of non-fermented WB. While the shear intensity increases, the apparent viscosity decreases until the viscosity reaches a plateau of limit viscosity, this pseudoplastic behavior is characteristic of WB (Dikeman et al., 2006). In contrast, as the fermentation time increases, WB/WP fermented by RW-9595M showed a lower viscosity than non-fermented WB and the other fermented samples. This behavior suggests different chemical interactions between the components of fermented WB/WP mixture (protein/polysaccharides) and EPS produced by RW-9595M (visible white pellicle, ropy strand formation) during fermentation. In this sense, the ratio of low-molecular-mass EPS may affect the viscosity of an EPS solution (Pham et al., 2000). The EPS fractions produced by *L. rhamnosus* E/N on a medium with galactose, lactose, or sucrose show heterogeneous molecular weight, while those produced on a medium with maltose and glucose show only high-molecular-weight EPS (Polak-Berecka et al., 2015). The same authors reported that the reduction of viscosity was correlated with the thickness of the EPS chain. The rheology of the EPS from *L. rhamnosus* RW-9595M, due to its acidic nature, may also be affected by electrostatic interactions between charged residues such as occurs with high-methoxyl pectin (Polak-Berecka et al., 2015; Bertsch et al., 2019).

### *In vitro* Digestion of Bioingredients

In this work, the macro-components such as dietary fiber, ash and total protein of fermented products did not differ from the non-fermented NFWB. In contrast, the bioingredients showed a lower content of the sugars lactose, galactose and xylose than NFWB, indicating the utilization of sugars from whey permeate or wheat bran (xylose) as a carbon source for growth of the strains. Furthermore, the content of water-soluble polysaccharides increased, principally in LR95C, LR11C, and LRWC in comparison to NFWB. The presence of the yeast possibly influenced the content of water-soluble polysaccharides of bio-ingredients after co-culture. The content of WSP includes EPS and non-hydrolyzed soluble polysaccharides retained by dialysis from wheat bran such as arabinoxylan, beta-glucans and yeast cell wall polysaccharides. In previous studies, co-culture with the yeast resulted in increased EPS production compared to monoculture by these three strains (49% for ATCC 9595; 42% RW-9595M, and 39% R0011) in WP medium (Bertsch et al., 2019). The more complex nature of the WB/WP matrix compared to WP alone could influence the production of EPS by RW-9595M. The reduction in viscosity observed during fermentation could be related to EPS heterogeneity of molecular mass distribution and the fraction of low molecular weight that cannot be retained by the dialysis membrane used for WSP quantification in this work. This may explain the slight differences of WSP content in BI from RW-9595M in mono and co-culture for a strain reported to produce high amounts of EPS.

Also as a result of fermentation, the content of soluble protein was lower, and the free amino acid profile was enriched in the bioingredients. The fermentation of WB/WP led to an increase in amino acids such Pro (49–77%) and Glu (46 to 68%), which could also partially originate from prolamin degradation. Arte et al. (2015) reported that primary proteolysis occurs when oligopeptides are released during cereal fermentation, mainly by the activity of cereal endoproteases. In fermented wheat bran or sourdough, microbial peptidase activity from lactic acid bacteria such as *L. rhamnosus* could be responsible for secondary proteolysis to release free amino acids and small-sized peptides (Coda et al., 2012). Accumulation of amino acids in dough during fermentation with lactobacilli depends on the strain (Gänzle et al., 2008). In this work, the co-culture revealed an enriched amino acid profile compared to monoculture. In this sense, the relative expression of the gene coding for dipeptidyl aminopeptidase (*R0011_04490*) was overexpressed in co-culture with yeast for these strains in a medium with WP (Bertsch et al., 2019). Further experiments would be necessary to determine how peptides and amino acids impact the bioactivity of bioingredients for use as antioxidants or their potential ACE-inhibitory activity due to peptide accumulation.

### Impact of Fermentation and *in vitro* Digestion on Bioactive Compounds and Antioxidant Activity

Fermentation had an effect on free and bound TPC and antioxidant activity of bioingredients. In general, the bound TPC and antioxidant activity of bioingredients were higher than the free fraction and the non-fermented samples. This agrees with previous reports revealing that fermentation improved the free and/or bound phenolic content of lentils, soy bean, black cow gram and mottled cowpea, wheat, rye and whole barley (Gan et al., 2016; Shumoy et al., 2017). The structural breakdown of the complex cereal cell wall induced by the endogenous enzymes of the cereals releases bioactive compounds. These enzymes are activated by the changes of pH during the fermentation process (Mateo Anson et al., 2011). Hence, fermentation could improve extraction efficiency of both free and bound phenolic compounds, leading to a higher TPC after processing (Gan et al., 2016). These TPC can act as reducing agents and singlet oxygen quenchers (Hur et al., 2014), thus increasing antioxidant activity.

In monoculture, the TPC (bound and total) and the antioxidant activity were higher than in co-culture for ATCC 9595 and especially for RW-9595M. Dordevic et al. (2010) also reported higher antioxidant activity in fermented buckwheat, wheat germ, barley and rye with *L. rhamnosus*, compared with samples fermented with *S. cerevisiae*. Hur et al. (2014) reported that lactic acid bacteria have antioxidant activity themselves, with mechanisms to reduce the impact of reactive oxygen species on cells. In addition, these three strains are EPS producers and these polysaccharides are synthesized in the WB/WP medium, as shown by scanning electron microscopy. In this sense, Polak-Berecka et al. (2015) reported antioxidant activity of the EPS produced by *L. rhamnosus* E/N, which produces EPS with the same structure as those produced by the strains used in this work.

Common groups of phenolic acids in cereals are hydroxybenzoic acid and hydroxycinnamic acid derivatives. Protocatechuate is one of the microbial degradation products of various aromatic compounds, including phytic acid, hydroxybenzoic acid, or hydroxycinnamic acid compounds such as ferulic acid. In a previous study, yeast co-culture with *L. rhamnosus* R0011, ATCC 9595 and principally RW-9595M showed an important increase of relative gene expression of carboxymuconolactone decarboxylase (*pcaC*) involved in the conversion of protocatechuate to 3-oxoadypate in co-culture with *S. cerevisiae* (Bertsch et al., 2019). In the β-ketoadipate pathway, 3-oxoadipate is then converted to succinyl-CoA and acetyl-CoA, which are processed by the tricarboxylic acid cycle (TCA) to release stored energy through the oxidation of acetyl-CoA into adenosine triphosphate (ATP) and carbon dioxide.

The increase in the amount of caffeic acid after fermentation of the bioingredients varied among the three strains (ATCC 9595 > RW-9595M > R0011) in both types of culture (mono or co-culture). The increase in free caffeic acid and the resulting decrease of ferulic acid or *p-*coumaric acid was also obtained for these strains of *L. rhamnosus* in a culture medium with WP and corn steep liquor (Bertsch et al., 2019). Possible mechanisms for microbial bioconversion could be (a) caffeic acid produced by hydroxylation of *p-*coumaric acid at the meta position similar to *Streptomyces caeruleus* (Sachan et al., 2006); (b) caffeic acid produced by the *o*-demethylation of ferulic acid. This pathway was shown in *Enterobacter cloacae* and *Clostridium methoxybenzovorans* and in human colonic fermentation of rye bran fortified bread (Micard et al., 2002; Filannino et al., 2014). Filannino et al. (2014) had confirmed the ability of some lactic acid bacteria such as *Lactobacillus* spp. to convert protocatechuic acid to catechol; *p-*coumaric to phloteric acid or *p-*vinylphenol. (c) chlorogenic acid may be transformed into either vynilcatechol, dihydrocaffeic or dihydroshikimic acid. Caffeic acid and shikimic acid appeared in the culture medium as intermediary products of the metabolism of phenolic acids (Sánchez-Maldonado et al., 2011). The enrichment with caffeic acid can have numerous beneficial properties: this phenolic acid has a stronger inhibition of *Staphylococcus aureus* (Stojković et al., 2013); colonic pathology and inflammation could be improved in mice with DSS colitis, and a proportional increase in *Akkermansia* may be associated (Zhang et al., 2016). Finally, caffeic acid may restrain the progression of type 2 diabetes through attenuating the output of hepatic glucose and enhancing uptake of adipocyte glucose, insulin secretion, and antioxidant capacity (Jung et al., 2006).

The total individual bound phenolic acid content increased after fermentation. Similarly, a previous study (Gan et al., 2016) reported that fermentation increased the bound phenolic acid contents of ferulic acid and *p-*coumaric acid in mottled cowpea. The individual content in bound phenolic acids was enhanced more from strain RW-9595M in co-culture compared with monoculture. In this study, bio-processing coupled with *in vitro* digestion resulted in higher TPC and antioxidant activity (ORAC), as found after *in vitro* digestion of bread made with buckwheat flour (Szawara-Nowak et al., 2016). The authors hypothesized that the TPC and antioxidant activity increase may be associated with pH and enzymatic interactions that occur during *in vitro* digestion resulting in the gradual release of total polyphenols during digestion. In contrast, the free individual phenolic acid content in digested samples was mainly solubilized during the simulated gastrointestinal process resulting in a decrease of their concentration in the non-soluble residue. The individual bound phenolic acid content was concentrated in the non-soluble residue, which might result in the appearance of higher TPC and antioxidant activity (ORAC) in the digested samples.

In order to evaluate the bioavailability of phenolic compounds for potential benefits, we determined the portion that was released from the food matrix, converted during digestion and accessible for absorption in the small intestine as well as the portion that undergoes degradation by the gut microbiota (unabsorbed) during gastrointestinal digestion. The quantity of recovered (unabsorbed) TPC and antioxidant activity remaining in the samples was highest in non-fermented NFWB. For all three strains, TPC recovery was higher from ingredients produced by co-culture than from those produced by monoculture, although the bioingredients obtained with *L. rhamnosus* RW-9595M showed the lowest percentage value of TPC recovered. Bio-ingredients from monoculture showed the highest release of TPC and antioxidant activity through *in vitro* digestion.

At the same time, the process enhanced the bioaccessibility of free phenolic acid compounds such as caffeic and 4-hydroxybenzoic acids. This is in accordance with Dall’Asta et al. (2016) who reported that in aleurone-enriched bread and whole-grain bread, sinapic and caffeic acid were better absorbed than ferulic acid or *p-*coumaric acids. One of the most abundant phenolic compounds in WB is ferulic acid, accounting for 90% of TPC in wheat grain (Mateo Anson et al., 2009). In WB, ferulic acid and its isomers represent aleurone and pericarp cell wall components, mainly esterified with arabinoxylans (Cömert and Gökmen, 2017). Fermentation of WB/WP provides added value for the production of new fermented cereal-based foods with enhanced bioaccessibility of free phenolic acids which is a prerequisite for their bioavailability.

Bioingredients derived from RW-9595M showed lower bioaccessibility of free ferulic acid compared with NFWB and the other bioingredients. On the other hand, the recovery of individual bound phenolic acids was higher from monoculture bioingredients compared to co-culture for strains R0011 and RW-9595M while no difference was found between mono and co-culture for strain ATCC 9595. The type of culture (mono or co-culture) of the three strains influenced the bioaccessibility of free phenolic acid and the recovery of TPC, antioxidant activity or bound phenolic acid of the bioingredients. Gullon et al. (2015) reported interaction with other diet compounds during digestion, such as iron, polysaccharides, other minerals or proteins as well as modification of chemical structure. Solubility changes are known to impact phenolic bioaccessibility and bioavailability. The chemical interactions between EPS or WSP from *L. rhamnosus* and phenolic acid compounds in WB/WP have not been elucidated to date. The EPS produced by these strains are acidic and could find applications where high-methoxyl pectin is used (van Calsteren et al., 2002). Phan et al. (2015) noted interactions of water-soluble phenolic compounds (phenolic acids, phenolic acid esters, flavan-3-ols, and anthocyanidins) with cellulose. Also, phenolic acids such as caffeic acid and ferulic acid interact with pectin. Bread fortified with high-methoxyl pectin and polyphenols from fruits showed a modification of protein structure exhibiting hydrophilic characteristics during processing via the formation of H bonds with water, polyphenols and polysaccharides (Sivam et al., 2013). These results are the first step to evaluating the effects of fermentation by EPS producer strains on the bioactivity of combined cereal/whey permeate bioingredients.

## Conclusion

The presence of free and bound phenolic compounds in bio-ingredients is an advantage in products which could act to control oxidative radical species (balance of soluble and insoluble antioxidant compounds) along the entire digestive tract (Cömert and Gökmen, 2017). The bound phenolic acid of fermented WB could reach the colon, where they would be converted by the gut microbiota into their metabolites such as 3-phenylpropionic acid (Mateo Anson et al., 2009). At the same time, phenolic compounds remaining after digestion may exert antioxidant action and antibacterial activity to limit pathogenic bacteria in the colon (Gong et al., 2018). In this sense, future experiments should involve the determination of colonic bioaccessibility of the residual bound phenolic compounds of these bioingredients and evaluate the potential prebiotic activity of foods containing EPS. Furthermore, the evaluation of functionality of BI should be done in order to reveal the impact of fermentation on the characteristic of EPS (molecular weight), rheology, water absorption of the mixture WB/WP to determine applications in sourdough. The research in this area appears to be a challenge, but promising, as results will have direct impact on the development of fermented dairy-cereal-containing functional foods to add value to wheat milling by-products.

## Data Availability Statement

The raw data supporting the conclusions of this article will be made available by the authors, without undue reservation, to any qualified researcher.

## Author Contributions

DR, AB, and GL: conceptualization and formal analysis. AB: methodology and writing – original draft preparation. GL and DR: resources, writing – review and editing, and funding acquisition. GL: data curation, supervision, and project administration. All authors contributed to the article and approved the submitted version.

## Conflict of Interest

The authors declare that the research was conducted in the absence of any commercial or financial relationships that could be construed as a potential conflict of interest.
