# Supplementary material for: Fermentation of Wheat Bran and Whey Permeate by Mono-Cultures of Lacticaseibacillus rhamnosus Strains and Co-culture With Yeast Enhances Bioactive Properties
Source: Front Bioeng Biotechnol. 2020 Aug 7;8:956. doi: 10.3389/fbioe.2020.00956 (PMC7427622; doi:10.3389/fbioe.2020.00956)
Supplement: Supplementary file 1 [file Table_1.docx]

**Electronic Supplementary Material Tables and Figures**

**
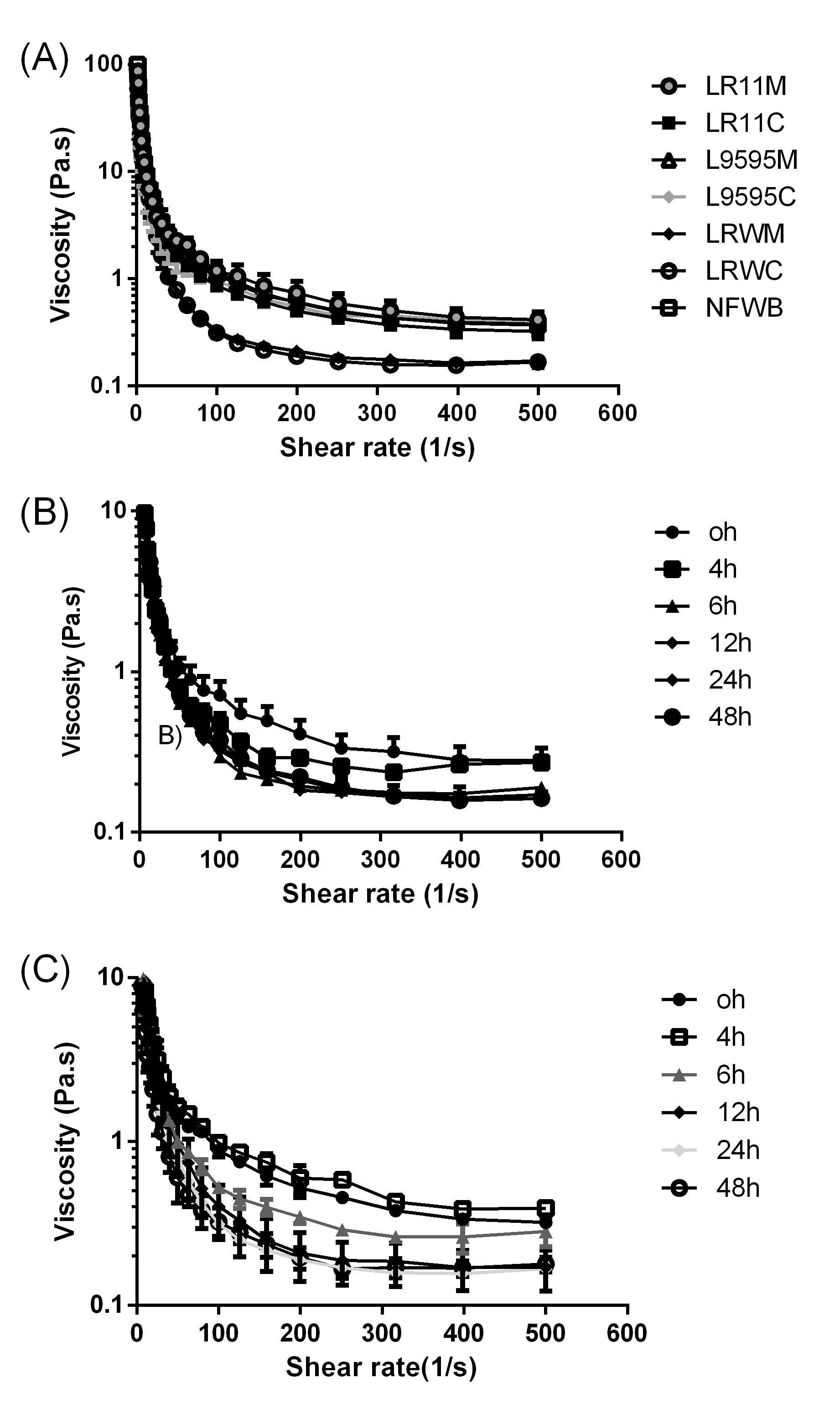
**

**Figure S1.** The viscosity flow curve for (A) the fermented substrates (F1-F6) or the unfermented wheat bran (WB) in 24h. WB/WP fermented by *Lacticaseibacillus rhamnosus* RW-9595M (0, 4, 6, 12, 24 and 48 h) in mono (B) or co-culture (C).


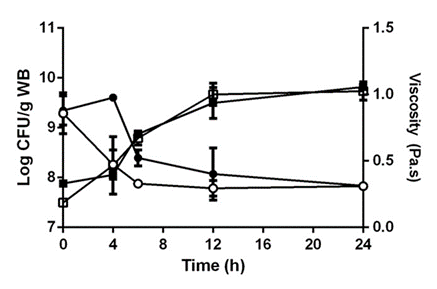


**Figure S2**. A) Microbial growth of RW-9595M (□ ■) vs apparent viscosity (Pa.s) (●○) at constant shear rate (100 s-1) after 24 h incubation in monoculture (black symbols) or co-culture with yeast (white symbols). (Pearson index r=-0.89 P=0.04 and r=-0.97 P=0.006 for mono and co-culture, respectively).


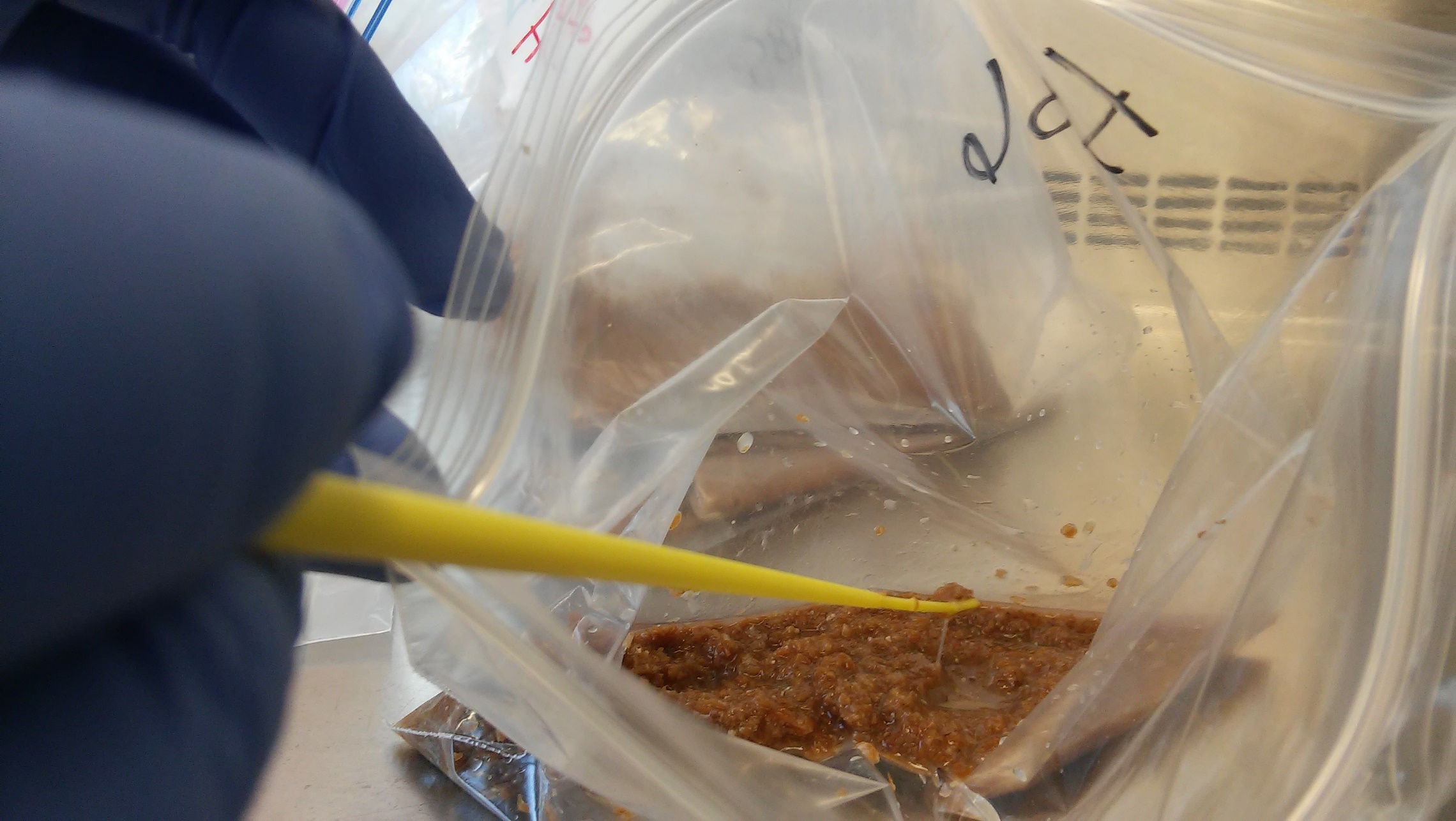


**Figure S3.** Macroscopic appearance of the “ropy” strand formed by the cellular mass of EPS-producing RW 9595-M in fermented wheat bran (BWF)


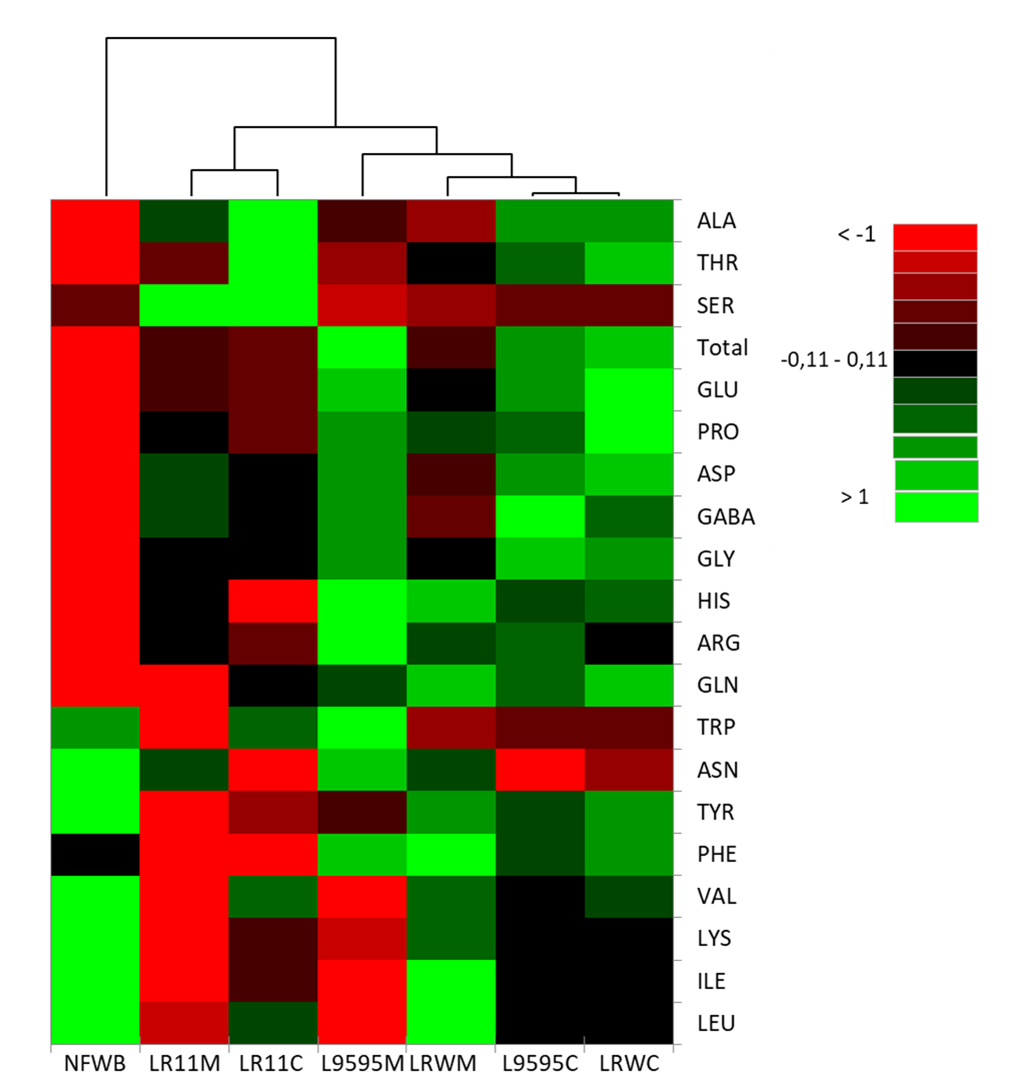


**Figure S4.** Heat map representation of free amino acid content of bioingredients and unfermented BWB substrate. The data was log-transformed. The heat map allow to visualize the result of hierarchical clustering of the profile data and to identify any positive or negative quantitative relationships (green and red, respectively) between bio-products and free amino acid content. Analysis of the heat map (dendrogram) distinguishes groups of BWB (unfermented) and the bioingredients (fermented). Thus, in fermented bioproducts, 63% of the amino acids were significantly higher (P<0.05) after bioprocessing (Arg, Gln, Ser, Gly, Thr, Ala, GABA, Pro, Asp, His, Glu, and Phe) in comparison to BWB.
